# A Novel Balance Control Strategy Based on Enhanced Stability Pyramid Index and Dynamic Movement Primitives for a Lower Limb Human-Exoskeleton System

**DOI:** 10.3389/fnbot.2021.751642

**Published:** 2021-11-25

**Authors:** Fashu Xu, Jing Qiu, Wenbo Yuan, Hong Cheng

**Affiliations:** ^1^School of Automation Engineering, University of Electronic Science and Technology of China, Chengdu, China; ^2^Machine Intelligence Institute, School of Automation Engineering, University of Electronic Science and Technology of China, Chengdu, China; ^3^Engineering Research Center of Human Robot Hybrid Intelligent Technologies and Systems, Ministry of Education, University of Electronic Science and Technology of China, Chengdu, China; ^4^Buffalo Robot Technology (Chengdu) Co., Ltd., Chengdu, China

**Keywords:** human-exoskeleton, enhanced stability pyramid, dynamic movement primitives, safety, gait planning, XCoM, SCI

## Abstract

The lower limb exoskeleton is playing an increasing role in enabling individuals with spinal cord injury (SCI) to stand upright, walk, turn, and so on. Hence, it is essential to maintain the balance of the human-exoskeleton system during movements. However, the balance of the human-exoskeleton system is challenging to maintain. There are no effective balance control strategies because most of them can only be used in a specific movement like walking or standing. Hence, the primary aim of the current study is to propose a balance control strategy to improve the balance of the human-exoskeleton system in dynamic movements. This study proposes a new safety index named Enhanced Stability Pyramid Index (ESPI), and a new balance control strategy is based on the ESPI and the Dynamic Movement Primitives (DMPs). To incorporate dynamic information of the system, the ESPI employs eXtrapolated Center of Mass (XCoM) instead of the center of mass (CoM). Meanwhile, Time-to-Contact (TTC), the urgency of safety, is used as an automatic weight assignment factor of ESPI instead of the traditional manual one. Then, the balance control strategy utilizing DMPs to generate the gait trajectory according to the scalar and vector values of the ESPI is proposed. Finally, the walking simulation in Gazebo and the experiments of the human-exoskeleton system verify the effectiveness of the index and balance control strategy.

## 1. Introduction

Spinal cord injury (SCI) is temporary or permanent damage to the spinal cord and might cause various impairments. The incidence of SCI is 40–80 new cases per 1,000,000 people per year from all causes, depending on the country (Fang et al., 2020). SCI will result in weak or paralyzed muscles, atrophy, walking disability, sensory dysfunction (Craggs et al., 2006), and autonomic disorders. Spasticity and pain are also some consequences of SCI affecting locomotor and quality of life (Barbeau et al., 1999). Also, the financial burden for rehabilitation is considerable. So, in recent years, the lower limb exoskeleton has gained considerable interest in improving the patients' life quality and reducing the financial burden since it could help the SCI patients with rehabilitation and walking assistance.

However, most patients cannot accept the exoskeleton quickly due to the security risk, like falling during walking. Moreover, once a fall occurs, it would cause secondary damage, causing the condition of the patient to worsen, even death (Brotherton et al., 2007). Therefore, it is necessary to establish a reasonable approach to accurately evaluate and perform the balance control of the safety of the human-exoskeleton system.

After summing up the research, it is found that there are three main types of methods that each use fundamentally different approaches for assessing the safety of the exoskeleton: 1) Biomechanical principles-based methods, like Stabilizing and Destabilizing Forces (SDF) (Duclos et al., 2009), Angular Momentum based Stability Index (AMSI) (Nott et al., 2013), Centroidal Momentum based Stability Index (CMSI) (Jung and Veneman, 2019), Foot Rotation Indicator (FRI) (Ali AbulKareem, 2020), Inverted Pendulum Model Approach (IPMA) (Elhasairi and Pechev, 2015), Capturability Region (CR) (Hong, 2019), Foot Placement Indicator (FPI) (Zutven et al., 2012); 2) Dynamic system theory-based methods, like Nearest Neighbor Gait Index (NNGI) (Gallego et al., 2012), Gait Sensitivity Norm (GSN) (Hobbelen and Wisse, 2007); 3) Probabilistic methods, like Trunk Orientation based Stability Index (TOSI) (Radkhah et al., 2010), Control Error Anomaly (CEA) (Ahmed and Ashton-Miller, 2007) and so on.

Stephens and Christopher presented a balance controller that allows a humanoid to recover from large disturbances and still maintain an upright posture in Stephens and Atkeson (2010). Balance is achieved by integral control, which decouples the dynamics and produces smooth torque signals. Chen et al. (2018) proposed a novel gait planning approach, which aims to provide reliable and balanced gait during walking assistance. This approach models the exoskeleton and patient together as a linear inverted pendulum (LIP) and obtains the intention of the patient through an orbital energy diagram. Guo et al. (2019) utilized a Zero Moment Point (ZMP) based method to obtain the center point position of the pressure and to get a mathematical expression on the stability of the human-machine system. By adjusting the pressure of the four support points of the two crutches and the feet of the exoskeleton robot, the system could tune the step sizes of the crutch and position dynamically to achieve the most stable motion state. Kopitzsch et al. (2017) presented an approach based on push recovery experiments while walking, multi-body system models, and least-squares optimal control to analyze the required torques to be generated by the exoskeleton, assuming that the human provides no torque. Wang et al. (2015) described the design, control, and preliminary evaluation of a novel exoskeleton, MINDWALKER. Besides powered hip flexion/extension and knee flexion/extension, it also has powered Hip Abduction/Adduction (HAA). In addition, a novel step-width adaptation algorithm was proposed to stabilize lateral balance. Current developments can only support people with paraplegia at most and require manual operation of crutches by the patient. To overcome this limitation, a theoretical model of a robotic device with actuated robotic crutches is proposed by Cohen and Or (2018), which can be used to support people with high-level disabilities, such as people with quadriplegia who cannot use the existing solutions to perform walking gaits. This study presents kinematic trajectory planning of the proposed model and dynamic analysis of main movement stages. Zhang et al. (2017) presented a high-power, self-balancing, passively and software-controlled active compliant, and wearable hip exoskeleton to provide walking and balance assistance. In addition, a new balance controller based on the “extrapolated center of mass" (XCoM) concept is presented for real-time control hip abduction/adduction to keep the center of mass (CoM) within the support polygon. The exoskeleton controller is designed to encourage participation in walking instead of overriding the intrinsic behavior users' to achieve practical assistance and training.

Most safety perception and balance control approaches cannot be applied to human-exoskeleton robots because they can only be used in a specific situation like walking or standing. Analyzing why people are not safe when wearing the exoskeleton, we summarized it for three reasons:

1. insufficient movement or perception (absence): After suffering from the SCI, they do not have enough capacity to control the exoskeleton to complete the desired action to prevent loss of balance.
2. not enough active degree of freedoms (DoFs): Though the lower limb exoskeletons could improve the motor ability of the pilot, most of them did not have enough DoFs, especially active ones, to keep the human-exoskeleton system safe.
3. insufficient cooperation between pilot and exoskeleton: The pilot has good safety perception and emergency handling ability, and the motor ability of the exoskeleton is good. Still, there is no practical way to communicate between them, especially the safety status.

According to the factors above, to keep the human-exoskeleton system safe, we must have a reliable safety indicator to tell both the pilot and the exoskeleton about the current safety status of the system. But so far, we could not find an effective index to complete this mission.

Among them, the Stability Pyramid Index (SPI) (Palani et al., 2018; Zhu et al., 2018) can indicate the safety of the whole system during all the movements of the human-exoskeleton system, but it also has some defects: 1) it is a static safety index that only involves the position of the CoM at that time; 2) the weight coefficient in the index is set manually without any theoretical basis; and 3) the index can only be expressed in scalars with limited information. Therefore, to improve its adaptability, we propose a new safety index named Enhanced Stability Pyramid Index (ESPI) for the exoskeleton with some obvious advantages:

1. it will represent the state of the system more fully and accurately by fusing the position, velocity, and acceleration of the human-exoskeleton system's CoM;
2. it will set the weight coefficient automatically with more interpretability and convenience by introducing the Time-to-Contact (TTC) in Slobounov et al. (1997) as the urgency of safety;
3. it will indicate the system's safety status and direction that is most likely to fall simultaneously with both scalar and vector.

With the help of the ESPI, a new balance control strategy for the human-exoskeleton system based on the Dynamic Movement Primitives (DMPs) is proposed. In this new balance control strategy, the scalar and vector values of ESPI could guide the spatial and temporal scaling features of DMPs. Then, the gait trajectory of the human-exoskeleton would be generated by it to keep the system's safety.

Therefore, this study makes a major contribution to research on the safety of the human-exoskeleton system by

1. proposing a new safety index named ESPI for the human-exoskeleton system;
2. promoting a balance control strategy combining ESPI and DMPs;
3. evaluating the index and control strategy with simulations and experiments.

This study is organized as follows: Firstly, the methodology of ESPI and the balance control strategy based on DMPs and ESPI will be described in detail in Section II. After introducing the human-exoskeleton system, the detailed model construction, data collection, and experimental verification of the ESPI and balance control strategy based on ESPI and DMPs are presented in Section III. In the last part, the summary of the proposed safety index, balance control strategy, and future study is demonstrated.

## 2. Methodology

This study proposes a novel balance control strategy for the human-exoskeleton system with a new safety index and DMPs. The ESPI was presented to indicate the safety status of the whole system. Simultaneously, it will consider the dynamic status of the human-exoskeleton system and provide more safety information *via* both scalar and vector.

**Figure 1:**
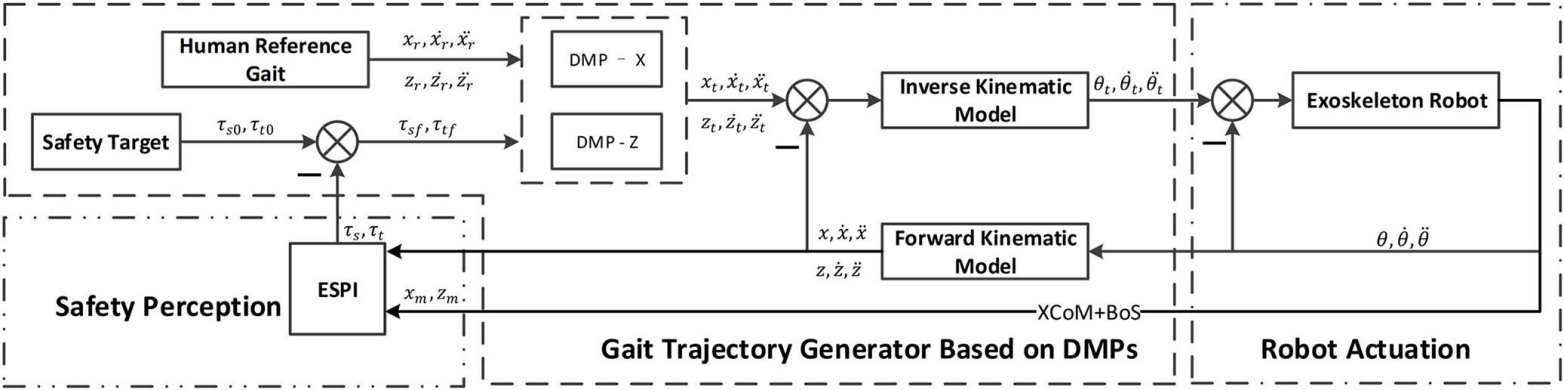
The architecture of the balance control strategy. When the XCoM, Base of Support (BoS), and the gait information of the human-exoskeleton system are collected, the scalar Φ and vector υ values of ESPI can be calculated, and the spatial and temporal scaling parameters (τ_*sf*_, τ_*tf*_) for the gait generator will be calculated. Once the scaling parameters are entered, the gait trajectory generator could plan a new gait based on dynamic movement primitives (DMPs) taking the standard gait as a reference. Additionally, joints trajectories generated by the inverse kinematic model could be executed by the exoskeleton robot to keep the system safe.

With the help of this index, the exoskeleton can sense the safety status of the human-exoskeleton system. Then, a new balance control strategy combining the ESPI with DMPs is built to eliminate unsafe factors according to tuned gait trajectories. The architecture of the balance control strategy shown in Figure 1 could be divided into three parts: Safety Perception, Gait Trajectory Generator Based on DMPs, and Robot Actuation.

In the control strategy, with the feedback of safety perception, the gait trajectory generator based on DMPs would tune the gait according to the relationships between the safety status and the gait. Also, the gait would be converted to joint trajectories, which could be executed by the joint servo drives.

In this section, we will describe the balance control strategy in detail according to its flow. So it comes to ESPI.

### 2.1. Enhanced Stability Pyramid Index

#### 2.1.1. Dynamic Status Modeling

In SPI, the vertex of the pyramid is the CoM of the system, and the contact points of the ground are taken as the corner points of the pyramid. For the human-exoskeleton system, the stability pyramid consists of the CoM and the contact points of the feet and sticks. The coordinate of CoM *P*_*CoM*_[*x*_*com*_, *y*_*com*_, *z*_*com*_] could be expressed as follows:


(1) $$\{\begin{array}{c} x_{com}=\sum_{i=1}^{k}\left(m_{i}x_{i}\right)/\sum_{i=1}^{k}m_{i}\\ y_{com}=\sum_{i=1}^{k}\left(m_{i}y_{i}\right)/\sum_{i=1}^{k}m_{i}\\ z_{com}=\sum_{i=1}^{k}\left(m_{i}z_{i}\right)/\sum_{i=1}^{k}m_{i} \end{array},$$

Among it, *x*_*i*_, *y*_*i*_, and *z*_*i*_ means the coordinate of *m*_*i*_, *k* is the total number of system parts. The CoM only contains the static position information, ignoring many dynamic details of the system. With the limited static position, the SPI could not fully picture the safety status of the entire system, especially in the human-exoskeleton system.

To incorporate the dynamic information of the system into ESPI, we replace the vertex of the stability pyramid with the XCoM (Hof et al., 2005) of the human-exoskeleton system. The coordinate of XCoM (*P*_*XCoM*_) is calculated based on *P*_*CoM*_ according to Equation (2).


(2) $$\begin{array}{l} \begin{array}{c} P_{XCoM}=P_{CoM}+\frac{V_{CoM}}{\omega_{0}}. \end{array} \end{array}$$

Additionally, the *P*_*XCoM*_[*x*_*xcom*_, *y*_*xcom*_, *z*_*xcom*_] would be:


(3) $$\{\begin{array}{c} x_{xcom}=x_{com}+\frac{{\dot{x}}_{com}}{\omega_{0}}\\ y_{xcom}=y_{com}+\frac{{\dot{y}}_{com}}{\omega_{0}}\\ z_{xcom}=z_{com}+\frac{{\dot{z}}_{com}}{\omega_{0}} \end{array},$$

where, $\omega_{0}=\sqrt{g/l}$, *l* means the length of the supporting leg or stick of the human-exoskeleton system, *g* is the acceleration of gravity, ẋ_*com*_, ẏ_*com*_, and ż_*com*_ are the velocity of CoM along the *x*, *y*, and *z* axis.

**Figure 2:**
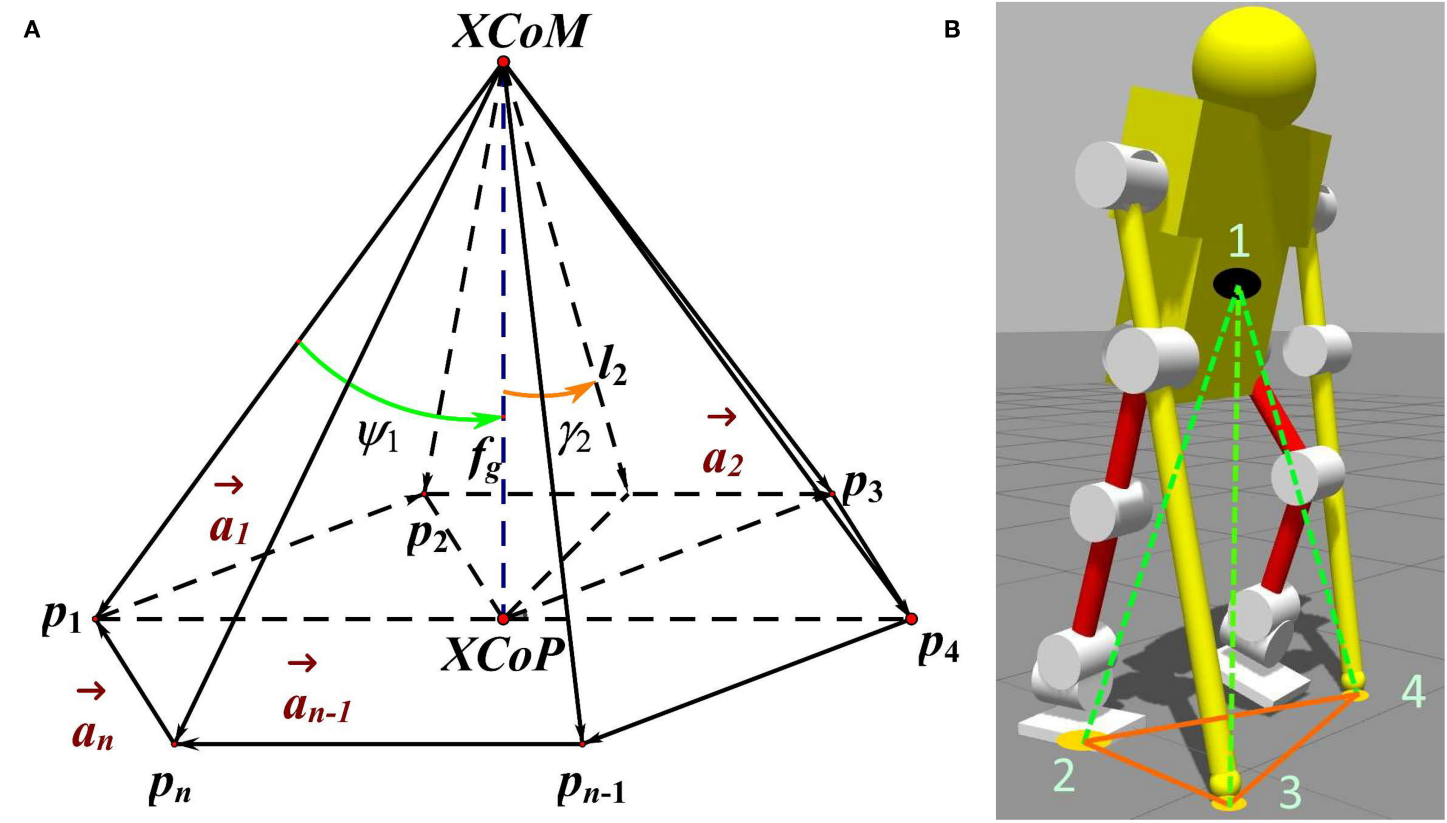
The Enhanced Stability Pyramid Index (ESPI) demonstration and the index of the human-exoskeleton model. In **(A)**, an enhanced stability pyramid is composed of the bottom points noted *p*_*i*_ and the vertex point—eXtrapolated Center of Mass (XCoM), which could be used to evaluate the safety status of a system. The stability angles over the edges and corners are marked as γ_*i*_ and ψ_*i*_ (*i* = 1, ⋯ , *n*). While in **(B)**, the pyramid of the human-exoskeleton to evaluate the system's safety is shown. In the pyramid, the label 1 means XCoM, and the points of contact with the ground are labeled with 2, 3, and 4.

In ESPI, the convex is *P*_*XCoM*_, the ground contact points of the system's feet and sticks are set as the bottom corner of the stability pyramid. The points are marked sequentially in a clockwise direction in a plan view, as shown in Figure 2A, defined as *p*_*i*_(*i* = 1, ···, *n*). They are a reference to the world coordinate system, the instantaneous position of each corner can be recorded as $p_{i}={\left[\begin{array}{ccc} p_{x} & p_{y} & p_{z} \end{array}\right]}_{i}^{T}$ and the polygon enclosed by them is called Base of Support (BoS). The stability pyramid of the human-exoskeleton system is shown in Figure 2B.

The edges of the stability pyramid's bottom surface marked as *a*_*i*_, the tilting edges, are defined as:


(4) $$\begin{array}{l} \begin{array}{c} a_{i}=p_{i+1}-p_{i}\;\left(i=1,\cdot \cdot \cdot ,n-1\right), \end{array} \end{array}$$

(5) $$\begin{array}{l} \begin{array}{c} a_{n}=p_{1}-p_{n}. \end{array} \end{array}$$

The straight lines *l*_*i*_ which are perpendicular to the tilting edge while passing the XCoM, are defined as:


(6) $$\begin{array}{l} \begin{array}{c} l_{i}=\left(I-{â}_{i}{â}_{i}^{T}\right)p_{i+1}, \end{array} \end{array}$$

in which, â_*i*_ = *a*_*i*_/∥*a*_*i*_∥ and *I* is a 3 × 3 unit matrix.

The stability angles over the edges of BOS γ_*i*_ can be defined as the angles between the perpendicular of the tilting edge *l*_*i*_ and the vector of gravity *f*_*g*_:


(7) $$\begin{array}{l} \begin{array}{c} \gamma_{i}=\sigma_{i}\cos^{-1}\left({\hat{f}}_{g}{\hat{l}}_{i}\right)\;\left(i=1,\cdot \cdot \cdot ,n\right), \end{array} \end{array}$$

where,


(8) $$\sigma_{i}=\{\begin{array}{l} +1,\;\left({\hat{l}}_{i}\times {\hat{f}}_{g}\right){\hat{a}}_{i}<0\\ -1,\;others \end{array},$$

(9) $$\begin{array}{l} \begin{array}{c} f_{g}=mg, \end{array} \end{array}$$

and *m* means the whole mass of human-exoskeleton system:


(10) $$\begin{array}{l} \begin{array}{c} m=\overset{k}{\underset{1}{\sum }}m_{i}. \end{array} \end{array}$$

Similarly, the stability angles over the corners of BOS ψ_*i*_ can be defined as the angles between the vector of gravity *f*_*g*_ and the lines passing through the corner points *p*_*i*_ and the XCoM.


(11) $$\begin{array}{l} \begin{array}{c} \psi_{i}=\varepsilon_{i}\cos^{-1}\left({\hat{f}}_{g}{\hat{p}}_{i}\right)\;\left(i=1,\cdot \cdot \cdot ,n\right), \end{array} \end{array}$$

in which,


(12) $$\varepsilon_{i}=\{\begin{array}{l} +1,\;\left({\hat{l}}_{i}\times {\hat{f}}_{g}\right){\hat{a}}_{i}<0\;or\;\left({\hat{l}}_{i+1}\times {\hat{f}}_{g}\right){\hat{a}}_{i+1}<0\\ -1,\;others \end{array}.$$

When it comes to the external forces marked as *f*_*ext*_, along every tilting axis, the equivalent force *f*_*i*_ can be expressed as:


(13) $$\begin{array}{l} \begin{array}{c} f_{i}=\left(1-{â}_{i}{â}_{i}^{T}\right)\left(f_{g}+f_{ext}\right). \end{array} \end{array}$$

The pilot in the human-exoskeleton system can move autonomously, so the stability index must consider the pilot's movement, and the acceleration of CoM caused by the pilot is defined as α. Therefore, the corresponding force is calculated as *f*_*a*_ = *ma*. So the equivalent force of the external forces *f*_*i*_ would be updated to:


(14) $$\begin{array}{l} \begin{array}{c} f_{i}=\left(1-{â}_{i}{â}_{i}^{T}\right)\left(f_{g}+f_{ext}+f_{a}\right). \end{array} \end{array}$$

If there is an external torque marked as τ_*ext*_ apart from the external forces, the equivalent force *f*_*i*_ would be updated as $f_{i}^{*}$ with an extra force item:


(15) $$\begin{array}{l} \begin{array}{c} f_{i}^{*}=f_{i}+\frac{{\hat{l}}_{i}\times \left({â}_{i}{â}_{i}^{T}\right)\tau_{ext}}{∥l_{i}∥}. \end{array} \end{array}$$

When considering the equivalent force, the stability angles of the tilting edges and corners need to be updated. The stability angles γ_*i*_ between the resultant force $f_{i}^{*}$ and the perpendicular of the tilting edge *l*_*i*_ could be updated as:


(16) $$\begin{array}{l} \begin{array}{c} \gamma_{i}=\sigma_{i}\cos^{-1}\left({\hat{f}}_{i}^{*}{\hat{l}}_{i}\right)\;\left(i=1,\cdot \cdot \cdot ,n\right), \end{array} \end{array}$$

where,


(17) $$\sigma_{i}=\{\begin{array}{l} +1,\;\left({\hat{l}}_{i}\times {\hat{f}}_{i}^{*}\right){\hat{a}}_{i}<0\\ -1,\;others \end{array},$$

(18) $$\begin{array}{l} \begin{array}{c} {\hat{f}}_{i}^{*}=\frac{f_{i}^{*}}{∥f_{i}^{*}∥}. \end{array} \end{array}$$

The stability angles ψ_*i*_ between the $f_{i}^{*}$ and the line passing through the corner point *p*_*i*_ and the XCoM could be updated as:


(19) $$\begin{array}{l} \begin{array}{c} \psi_{i}=\varepsilon_{i}\cos^{-1}\left({\hat{f}}_{i}^{*}{\hat{p}}_{i}\right)\;\left(i=1,\cdot \cdot \cdot ,n\right), \end{array} \end{array}$$

in which,


(20) $$\varepsilon_{i}=\{\begin{array}{l} +1,\;\left({\hat{l}}_{i}\times {\hat{f}}_{i}^{*}\right){\hat{a}}_{i}<0\;or\;\left({\hat{l}}_{i+1}\times {\hat{f}}_{i}^{*}\right){\hat{a}}_{i+1}<0\\ -1,\;others \end{array},$$

(21) $$\begin{array}{l} \begin{array}{c} {\hat{p}}_{i}=\frac{p_{i}}{∥p_{i}∥}. \end{array} \end{array}$$

To evaluate the global stability of human-exoskeleton system, the minimal stability angle α could be defined:


(22) $$\begin{array}{l} \begin{array}{c} \alpha =\min\left(\gamma_{1},\cdots \;,\gamma_{n},\psi_{1},\cdots \;,\psi_{n}\right). \end{array} \end{array}$$

When α < 0, the equivalent force at the CoM is out of the BoS of the stability pyramid, the human-exoskeleton system would tip over. When α = 0, the equivalent force is on the BoS side of the stability pyramid, the human-exoskeleton system is in a critical safe state. But owing to the errors of measurement and calculation, the system would be unbalanced. When α > 0, the equivalent force would keep the CoM in the BoS of the stability pyramid, which means the human-exoskeleton system would be balanced. In summary, α implies the stability of the system. The bigger it is, the safer the system is. So during the whole moving process, the most important thing is to find a maximal stability angle to keep the system balanced.

The minimum stability angle under static and dynamic conditions can be calculated by the above method. For system safety under dynamic conditions, the minimum tipping energy *E*_*t*_ also needs to be considered, which is the minimum energy consumed by the system to roll over the edge or corner point under dynamic conditions:


(23) $$\begin{array}{l} \begin{array}{c} E_{t}=Gh\left(1-\cos\alpha \right), \end{array} \end{array}$$

and the currently existing gravitational potential energy *E*_*g*_ could be expressed as follows:


(24) $$\begin{array}{l} \begin{array}{c} E_{g}=Gh\cos\alpha , \end{array} \end{array}$$

*h* is the distance between the vertex and the bottom of the stability pyramid, the height when the ground is a plane, *G* is the gravity of the human-exoskeleton system.

After analyzing the data, we found that when α > 0, the greater the ratio of *E*_*g*_ to *E*_*t*_ is, the safer the system is. So, we simplify it to a dimensionless expression further:


(25) $$\begin{array}{l} \begin{array}{c} \rho =\left(1-\cos\alpha \right)/\cos\alpha , \end{array} \end{array}$$

Finally, with the stability angles over the tilting edges and the corners, the prototype of ESPI Φ could be defined as follows:


(26) $$\begin{array}{l} \begin{array}{c} \Phi =\max\left(\frac{\zeta_{i}}{\gamma_{i}}+\frac{\eta_{i}}{\psi_{i}}+\frac{\sigma }{\rho }\right)\;\left(i=1,\cdot \cdot \cdot ,n\right), \end{array} \end{array}$$

in which, ζ_*i*_, η_*i*_, and σ are weighting coefficients of γ_*i*_, ψ_*i*_, and ρ, respectively. For the stability angles over the edges and corners of the stability pyramid, the smaller the angles are, the greater the possibility of tipping and the greater the weight value.

In this index, the CoM is replaced by the XCoM to introduce the dynamic state of the system, providing a more accurate understanding of the current dynamic safety situation of the system. Still, we need a more intelligent weighting factor than the manual one that can adjust automatically according to the urgency of the system's safety status.

#### 2.1.2. Urgency of Safety Modeling

To make the weighting factors more intelligent, we employ a new factor TTC, to automatically calculate the weights. TTC is the ability to estimate the time remaining before something reaches a person or a particular place. Additionally, it means the time it would take the XCoP to contact the BoS given its current trajectory. To evaluate the system status more accurately, the time before the XCoP crosses the BoS can be defined as:


(27) $$\begin{array}{l} \begin{array}{c} b_{\tau }=\frac{b}{V_{CoM}}, \end{array} \end{array}$$

in which, *b* is the distance between the XCoP and the boundary of BoS, *V*_*CoM*_ means the velocity of CoM. In addition, *b*_τ_, the time to go over BOS, could be used to express the urgency of safety.

In this study, the most important information may not be the current position of CoM or XCoM. Still, when it is unsafe in the future, which could be indicated as the urgency of safety, and *b*_τ_ could represent this parameter exactly. This parameter plays an important role: During this time, the safety status of the human-exoskeleton system may change with the current dynamic status. Also, with the movements of the pilot's arm and trunk, even postural control strategies, some CoM position and velocity corrections can be planned or executed to restore the system's safety.

So, the urgency of safety parameters could be defined as *b*_τ*γi*_ and *b*_τ*ψi*_ for the edges and corners of the BOS, respectively. When the *V*_*com*_ does not intersect with the edges or corners of the BoS, the variable *b*_τ*γi*_ and *b*_τ*ψi*_ would be infinity. To avoid that the weight value is 0 under any circumstances, the weights for the stability angles are defined as $\left(1+\frac{1}{b_{\tau \gamma i}}\right)$ and $\left(1+\frac{1}{b_{\tau \psi i}}\right)$. After merging the urgency of safety, the stability index Φ could be updated to:


(28) $$\begin{array}{l} \Phi =\max(\frac{1}{\gamma_{i}}(1+\frac{1}{b_{\tau \gamma i}})+\frac{1}{\psi_{i}}(1+\frac{1}{b_{\tau \psi i}})+\\ \;\frac{1}{\rho }(1+\frac{2}{b_{\tau \gamma i}+b_{\tau \psi i}})(i=1,\cdots ,n). \end{array}$$

The index Φ could represent the current safety status and the future safety trend based on the static and dynamic information of the human-exoskeleton system. So, when applying the index Φ into postural control, the current and future safety status could be considered simultaneously, which would help achieve better control.

#### 2.1.3. Scalar and Vector Representation

According to Equation (28), the scalar value Φ of ESPI can be calculated. The smaller the scalar is, the safer the system would be. During movement, the human-exoskeleton system should take various actions to make the index as small as possible to ensure its safety. To feedback more safety status information, besides the scalar Φ, another vector υ is introduced to characterize the change direction of the current system's safety, defined as *V*_*CoM*_.


(29) $$\begin{array}{l} \begin{array}{c} \upsilon =V_{CoM} \end{array} \end{array}$$

With the help of Φ and υ, the human-exoskeleton system's static and dynamic status would be represented more comprehensively, which could help the pilot and exoskeleton sense safety better. So, it comes to a balance control strategy based on it and DMPs.

### 2.2. Balance Control Strategy

#### 2.2.1. Dynamic Movement Primitives

After we get the safety status according to the safety perception approach, the gait trajectory for balance control needs to be generated to guide the exoskeleton to keep the system safe. While the pilot is patient with SCI, the trajectory of the exoskeleton must be similar to a human's. That is what the gait trajectory generator based on DMPs does. DMPs are a method of trajectory control/planning from the lab of Stefan Schaal. They were presented by Ijspeert et al. (2002), and then updated (Ijspeert et al., 2013). This study was motivated by the desire to find a way to represent complex motor actions that could be flexibly adjusted without manual parameter tuning or having to worry about instability. DMPs are used to generate discrete and rhythmic movements. The base system is a point attractor for discrete movements, and for rhythmic movements, a limited cycle is used. In this study, we focus on discrete movements of DMPs. According to Pastor et al. (2011), the original DMPs system is introduced as follows:


(30) $$\begin{array}{l} \begin{array}{c} \tau^{2}\dot{\upsilon }=K\left(g-x\right)-Dv-K\left(g-x_{0}\right)s+Kf\left(s\right) \end{array} \end{array}$$

(31) $$\begin{array}{l} \begin{array}{c} \tau ẋ=\upsilon \end{array} \end{array}$$

where *x* and *v* are position and velocity of the system; τ is a temporal scaling factor; *x*_0_ and *g* are the start and goal positions; *K* is the elasticity coefficient of the system; *D* is the damping coefficient of the system in a critical state; *f* is the nonlinear function to generate arbitrarily complex curves; *s* is the canonical dynamical system. The differential Equation (31) above indicates a transformation system. Among them, the nonlinear function *f* can be defined as:


(32) $$\begin{array}{l} \begin{array}{c} f\left(s\right)=\frac{\Sigma_{i}\omega_{i}\psi_{i}\left(s\right)}{\Sigma_{i}\psi_{i}\left(s\right)}s \end{array} \end{array}$$

where ψ_*i*_(*s*) is the basic Gaussian function, it can be defined as:


(33) $$\begin{array}{l} \begin{array}{c} \psi_{i}\left(s\right)=\exp\left(-h_{i}{\left(s-c_{i}\right)}^{2}\right) \end{array} \end{array}$$

in which, *c*_*i*_ is the center of the function, *h*_*i*_ is the height, and *w*_*i*_ is the weight of each Gaussian function.

From Equation (32), the Gaussian function *f* is not directly dependent on time parameters, but the phase parameter *s*, can be expressed as:


(34) $$\begin{array}{l} \begin{array}{c} \tau ṡ=-\alpha s \end{array} \end{array}$$

where α can be any constant. As can be seen from Equation (34), *s* decreases from 1 to 0 monotonically, that is called the canonical dynamical system. For a given α and τ, the corresponding parameter value *s* can be calculated, so the *f*_*target*_ in Equation (30) can be expressed as below:


(35) $$\begin{array}{l} \begin{array}{c} f_{target}\left(s\right)=\frac{\tau \dot{\upsilon }+D\upsilon }{K}-\left(g-x\right)+\left(g-x_{0}\right)s \end{array} \end{array}$$

To find the optimal weight value *w*_*i*_ in Equation (32), the regression algorithm was used to minimize (Equation 36; Pastor et al., 2011). With these parameters above brought into Equations (30) and (31), the position *x*, speed *v*, and acceleration $\dot{v}$ of the trajectory will be obtained.


(36) $$\begin{array}{l} \begin{array}{c} J=\Sigma_{s}{\left(f_{target}\left(s\right)-f\left(s\right)\right)}^{2} \end{array} \end{array}$$

**Figure 3:**
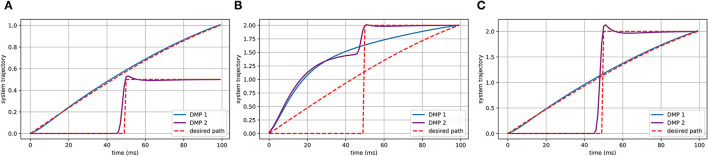
The spatial scaling feature of DMPs. The goals in **(A)** are 1 and 0.5, which are where the DMPs end up. When we want to get a scaled up version of this trajectory to a goal of 2 by DMPs, if the forcing function, with (*g* − *y*_0_), then we will end up with this shown in **(B)**. Once the (*g* − *y*_0_) term included in the forcing function, however, we get the **(C)** which is exactly what we want.

In Figure 3, the spatial and temporal scaling features of DMPs will be utilized to tune the gait based on the safety status. Spatial scaling means that once we have set up the system to follow the desired trajectory to a specific goal, we would like to be able to move that goal farther away or closer in and get a scaled version of our trajectory. This is what the (*g* − *y*_0_) term of the forcing function handles by scaling the activation of each of these basis functions relative to the distance to the target, causing the system to cover more or less distance.

**Figure 4:**
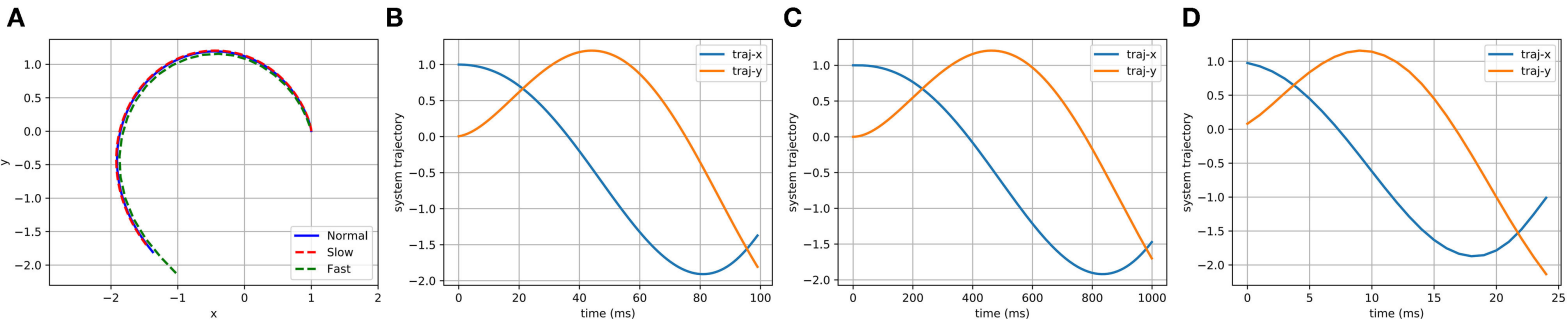
The temporal scaling feature of DMPs. In **(A)**, we can see that DMPs have planned three different trajectory speeds labeled as “Normal,” “Slow,” and “Fast,” which are shown in **(B–D)**. When comparing **(B–D)**, it is evident that the trajectories of the x-axis and y-axis are same, but the time to finish the path is different.

In the temporal case, we would like to be able to follow this same trajectory at different speeds, represented in Figure 4. Sometimes quick, sometimes slow, but always tracing the same path. To do that, we will add another term to our system dynamics, τ, our temporal scaling term. To give it temporal feasibility, we can add the τ term in Equation (31) and τ^2^ for $\dot{\upsilon }$ in Equation (30) because it is the second derivative that is. To slow down the system, you can set τ greater than 1; to speed it up, τ could be set between 0 and 1.

#### 2.2.2. Flow of Balance Control Strategy

Using the VICON motion capture devices during normal walking, the joint angle trajectories of the hip and knee can be obtained. Additionally, the normalized gait trajectory could be the initial one for DMPs to learn. With the safety perception parameters, the gait trajectory generator based on DMPs will generate different trajectories to walk safely, which is shown in Figure 1.

**Figure 5:**
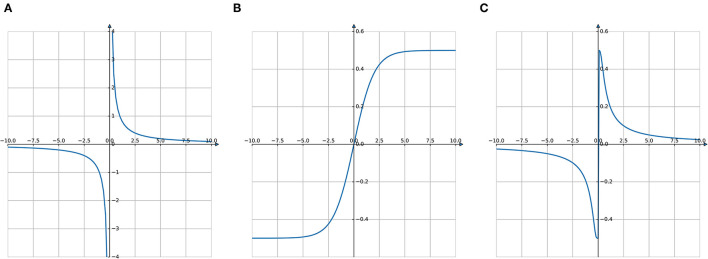
The relationships between safety status and the spatial scaling parameters. In **(A)**, the horizontal axis is the scalar value of the ESPI, and the vertical axis shows the safety status of the human-exoskeleton system. The larger the value of the vertical axis, the safer the system. In **(B)**, the horizontal axis is the safety status of the system, and the vertical axis shows the spatial scaling parameter τ_*s*_ for the gait trajectory generator based on DMPs. In **(C)**, the horizontal axis is the scalar value of the ESPI, and the vertical axis shows the spatial scaling parameter τ_*s*_ for the gait trajectory generator based on DMPs.

The ESPI would indicate the safety status of the system by the scalar and vector value in safety perception. The scalar value represents the current safety status of the system, while the vector would express the direction of the most drastic safety change in the future. The scalar and vector values should be converted to the spatial and temporal scaling parameters (τ_*s*_, τ_*t*_) for the gait generator to pass on the safety status to the next part. The relationships could be shown as the Equation (37) in Figure 5.


(37) $$\{\begin{array}{l} \tau_{s}=1/(1+e^{-1/\Phi })-0.5\\ \tau_{t}=1/\upsilon_{x} \end{array}$$

The spatial and temporal scaling parameters for the safety target defined in Figure 1 are τ_*s*0_, τ_*t*0_, while the parameters for the inputs of DMPs are τ_*sf*_, τ_*tf*_. According to the reference human gait, the gait trajectory generator based on DMPs will produce new gaits corresponding to different spatial and temporal scaling parameters τ_*sf*_, τ_*tf*_. The spatial and temporal parameters will change the stride length and stride time, respectively. The new gait will be converted to the joint angle trajectories *via* forward kinematics, and the exoskeleton robot could execute the motions of joints.

However, since the movements of the pilot of the human-exoskeleton system are uncontrolled, the closed-loop stability of the proposed control strategy is an open issue. It is a negative feedback control system; but, the human-exoskeleton system could fall directly when the movements of the upper body or the sticks are too large. Though the stability of the control strategy is open, there are two closed-loop stable feedback control systems in the human-exoskeleton system. One is the execution system of the gait in the gait generator. The newly generated gait could be transformed to joint trajectories by the inverse kinematic model to the joints. With the trajectories being executed, the encoders will update the joint angles of the exoskeleton. Finally, the gait executed by the exoskeleton could be the negative feedback transformed by the forward kinematic model, which could ensure the stability of this execution system. The other is the robot actuation control system in the exoskeleton. The planned joint trajectories are the inputs, and the joint servos could execute them. Contrary to open-loop systems, closed-loop motor controls in joint servo are designed to automatically achieve the target output condition and maintain it by feeding back the actual state of the motor, such as torque, velocity, and position.

## 3. Experiments

### 3.1. Simulation Environment

The feasibility of the proposed stability index ESPI has been verified efficiently and safely using a human-exoskeleton model and joint controller, based on the Robot Operating System (ROS) and Gazebo. With the balance control method of a LIP, the human-exoskeleton model could walk continually without falling.

**Figure 6:**
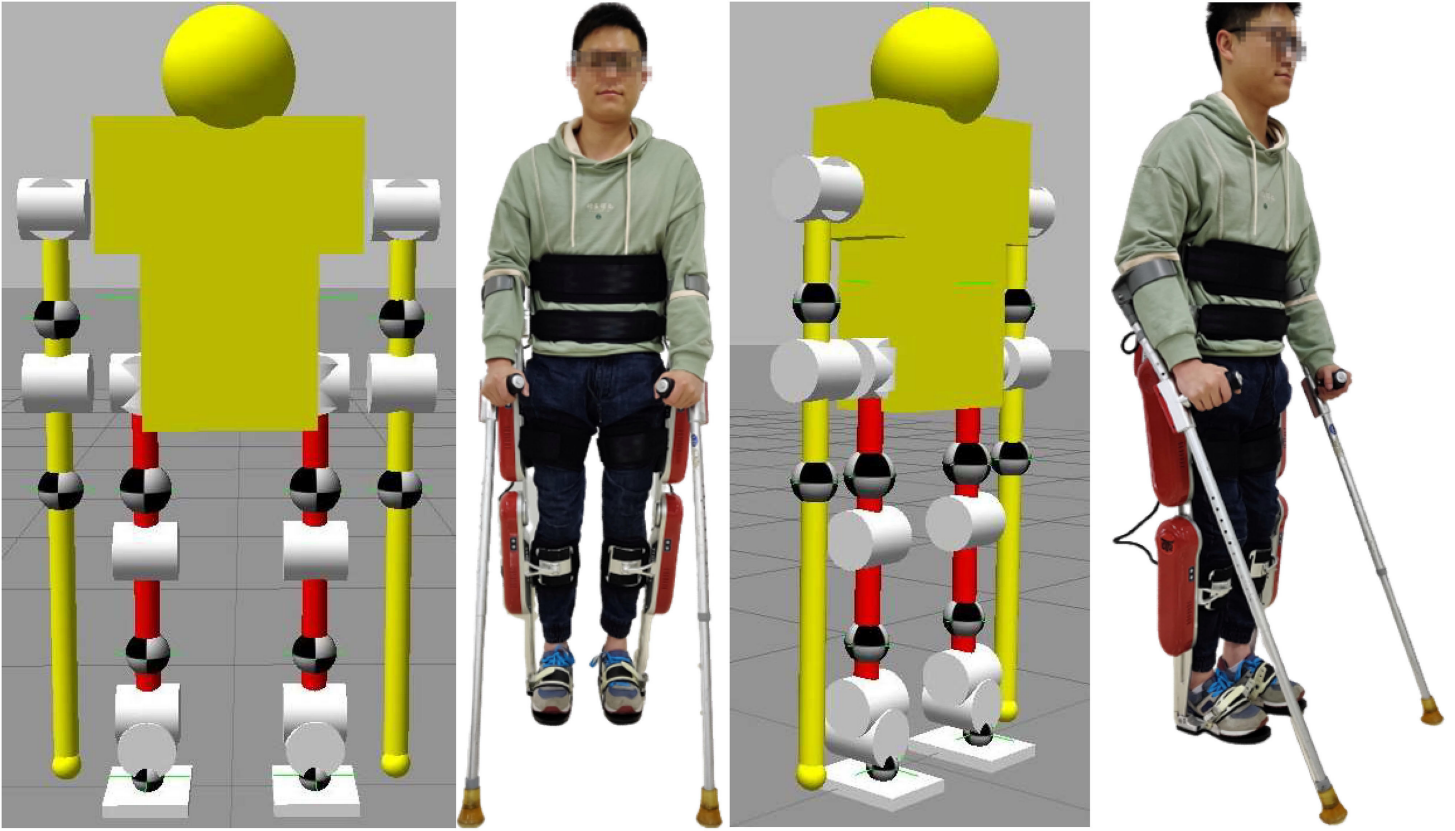
The front and side of the human-exoskeleton model. This human-exoskeleton model has 10 active joints and 16 active degree of freedoms (DoFs), namely the left and right shoulder, wrist, hip, knee and ankle joints. In addition, the shoulder, hip and ankle joints on the left and right have two active DoFs, while the others only have one.

The pilot and exoskeleton need to help each other complete the movements, making them a new human-exoskeleton system. As shown in Figure 6, this model has 10 active joints and 16 active DoFs, namely shoulder, wrist, hip, knee and ankle joints on the left and right. Among them, the left and right shoulder, hip, and ankle joints have two active DoFs, while the others only have one. The model is designed based on the dynamics of the human-exoskeleton system and shows the position of the center of gravity of each segment.

**Figure 7:**
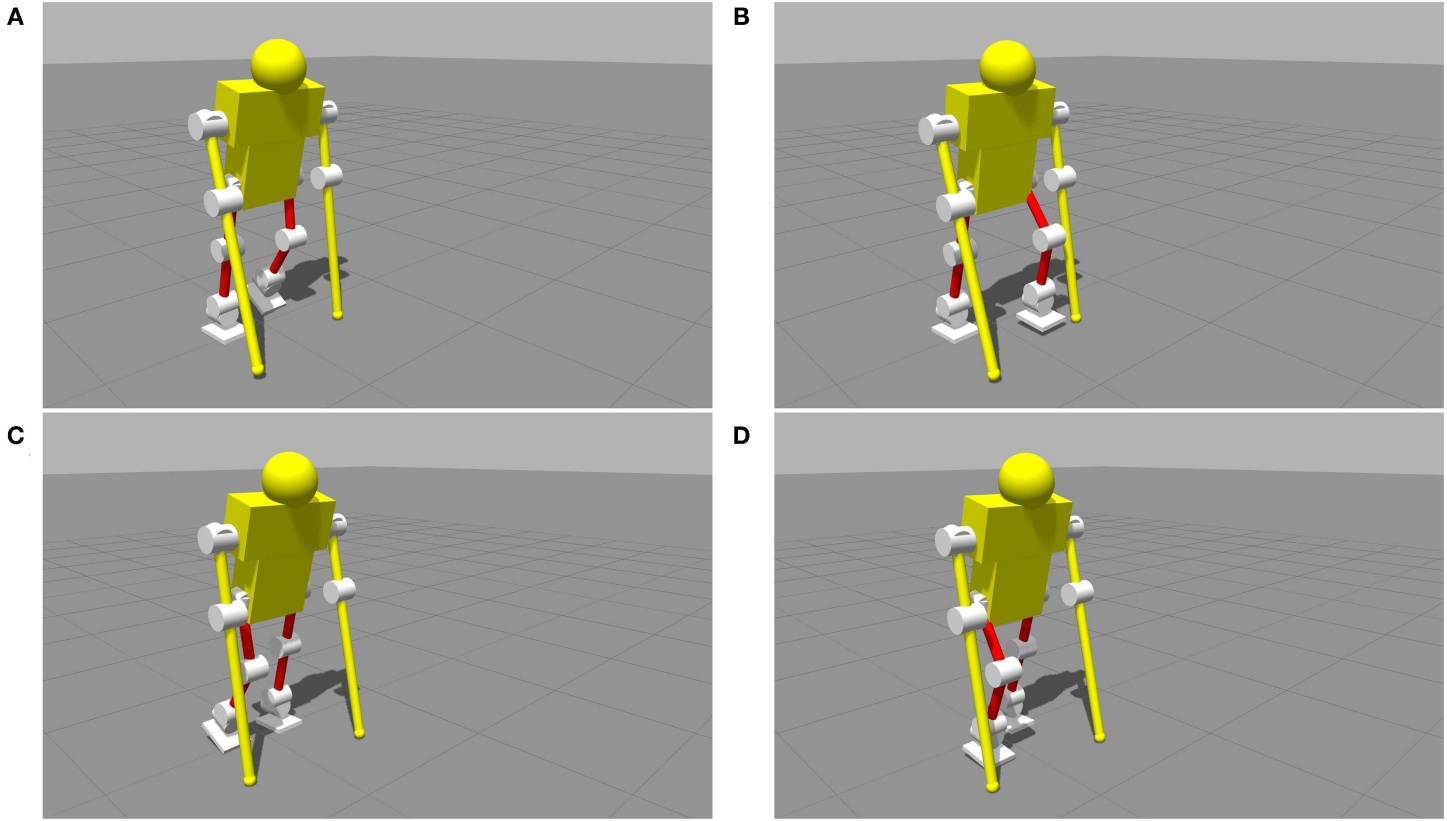
The simulation of a smooth walk with the linear inverted pendulum (LIP) method. These figures represent a complete gait cycle, starting from the left foot to the right foot, shown in **(A–D)** in turn.

To get the position, velocity, and acceleration information of the CoM, the Statically Equivalent Serial Chain (SESC) (Cotton et al., 2009; Bonnet et al., 2015) would be used. With the LIP control method (Kajita et al., 2001) and the sensor information, this model could walk smoothly forward all the time as shown in Figure 7 with the raw sensor data of ESPI being recorded.

### 3.2. Simulation Experiments

During the walking simulation process of the human-exoskeleton system, all data to calculate ESPI, including the position, velocity, and acceleration of the CoM, the contact position of the feet and sticks, are collected. They are referenced to the world coordinate system in the simulation environment. Also, the human-exoskeleton system would have 1–4 contact points with the ground. Since 1–2 contact points cannot maintain a BoS and cannot maintain balance for a long time, we will exclude these data and only focus on the situation of 3–4 contact points in the analysis process of this experiment.

**Figure 8:**
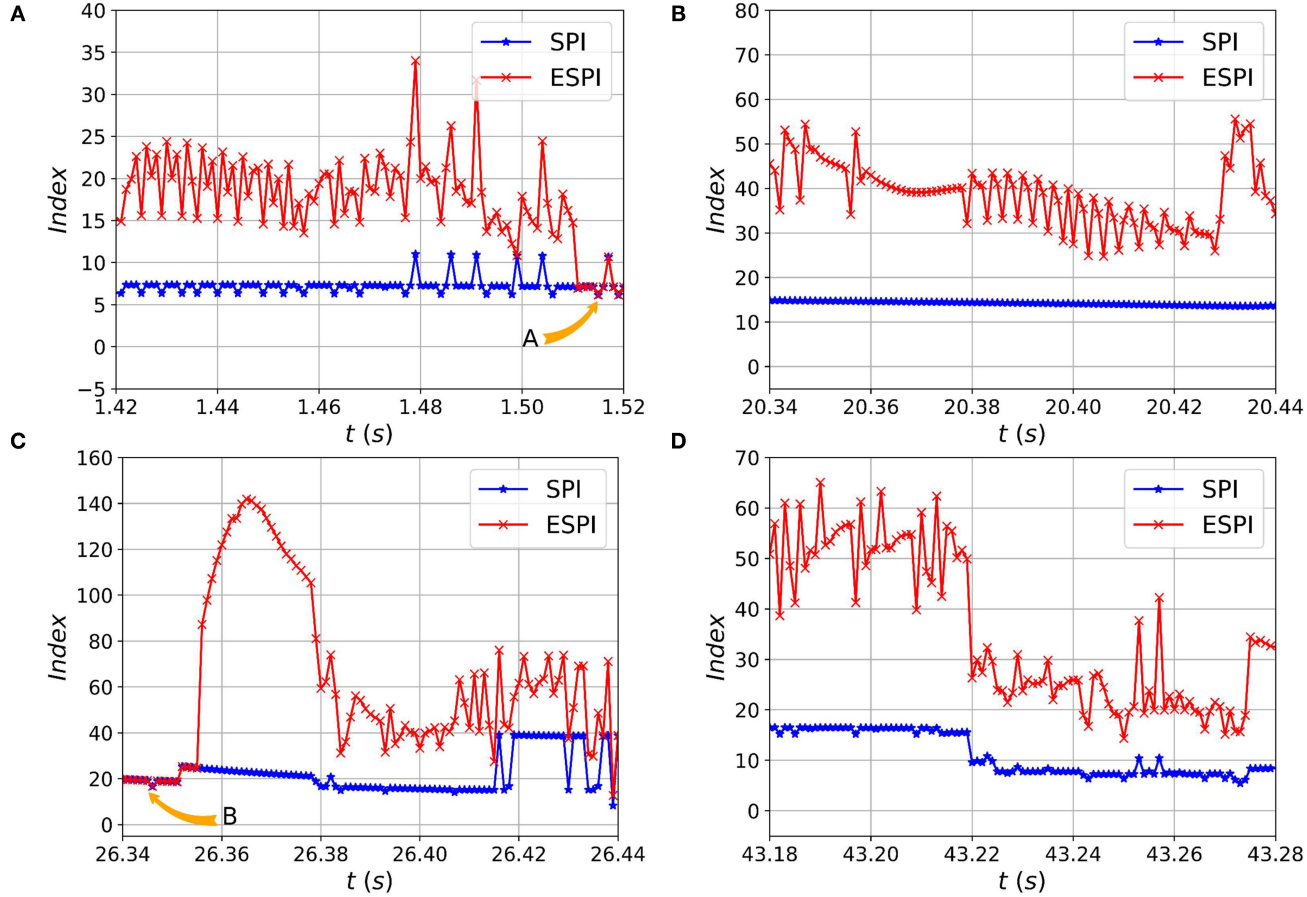
The comparison of ESPI and Stability Pyramid Index (SPI) during the walking experiments. The red and blue lines and dots represent ESPI and SPI obtained from the preliminary analysis at different times. The system is safe when SPI is less than 40, while ESPI is less than 150. The data in **(A–D)** shows that the ESPI is more sensitive than SPI, and they are the same when the CoM's velocity is zero labelled A and B in **(A,C)** respectively.

Also, the SPI is calculated according to Equation 26 with the weight coefficients ζ_*i*_, η_*i*_, and σ set to 1. Meanwhile, according to Equations 27 and 28, the ESPI and TTC are calculated during simulation. The comparison charts of ESPI and SPI are shown in Figure 8.

As shown from Figure 8, the human-exoskeleton system is in a safe walking state, which means the two indicators marked in the figure can accurately reflect the stability of the system. Both indicators are fluctuating during walking, but the fluctuation range of ESPI is significantly greater than that of SPI. It means that ESPI is more sensitive and effective than SPI to indicate the security status of the system, especially in Figures 8B,C. Sometimes, the value of ESPI and SPI are the same, which shows that the CoM of the system reaches the minimum kinetic energy and the maximum potential energy under the control of the LIP algorithm, as shown in Figures 8A,C labeled as *A* and *B*, respectively. But in most cases, the ESPI value is greater than the SPI, and the reason is that the CoM has a variable speed, which also proves the ESPI is more sensitive than the SPI.

**Figure 9:**
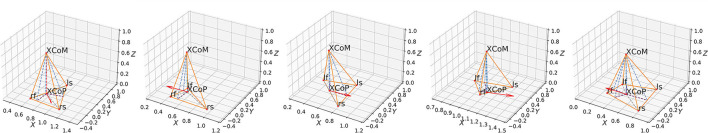
Five different types of stability pyramids with the safety change direction vector in ESPI are represented as *T*1, *T*2, *T*3, *T*4, and *T*5, respectively. Different combinations of contact points abbreviated as *lf*, *rf*, *ls*, and *rs* could make different types of stability pyramids with the XCoM as the vertex. Meanwhile, the vectors marked as the red arrow above mean the safety change direction.

**Figure 10:**
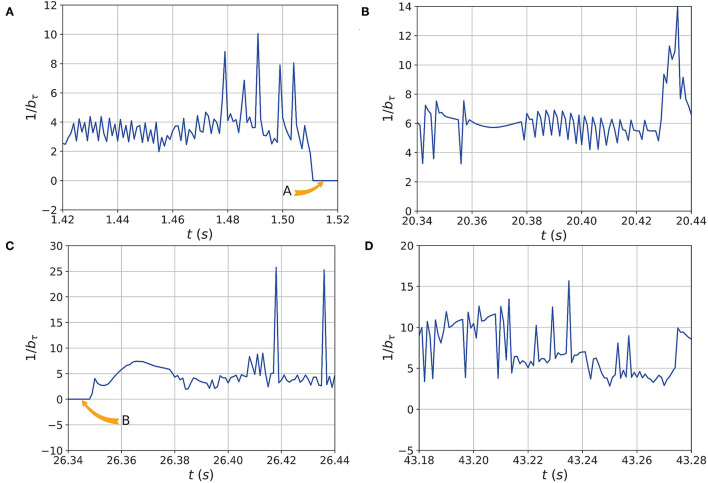
The urgency of safety based on the minimal Time-to-Contact (TTC) of the edges and corners of the BoS. As the velocity of CoM changes, the TTC will also vary as shown in **(A–D)**. When the velocity becomes 0, *b*_τ_ becomes infinity, and the urgency of safety will be 0 as shown in **(A,C)** marked as *A* and *B* respectively. This is exactly consistent with the results of the index shown in Figures 8A,C.

In the simulation, the system takes the XCoM as the vertex and forms five types of stability pyramid expressed as *T*1, *T*2, *T*3, *T*4, and *T*5 with different combinations of contact points shown in the Table 1. All stability pyramid are represented in Figure 9. And the urgency of safety is represented in Figure 10.

**Table 1:** Five different combinations of contact points.

	**Left foot**	**Right foot**	**Left stick**	**Right stick**
T1	✓	✓	✓	✓
T2		✓	✓	✓
T3	✓		✓	✓
T4	✓	✓		✓
T5	✓	✓	✓	

### 3.3. Human-Exoskeleton Experiments

**Figure 11:**
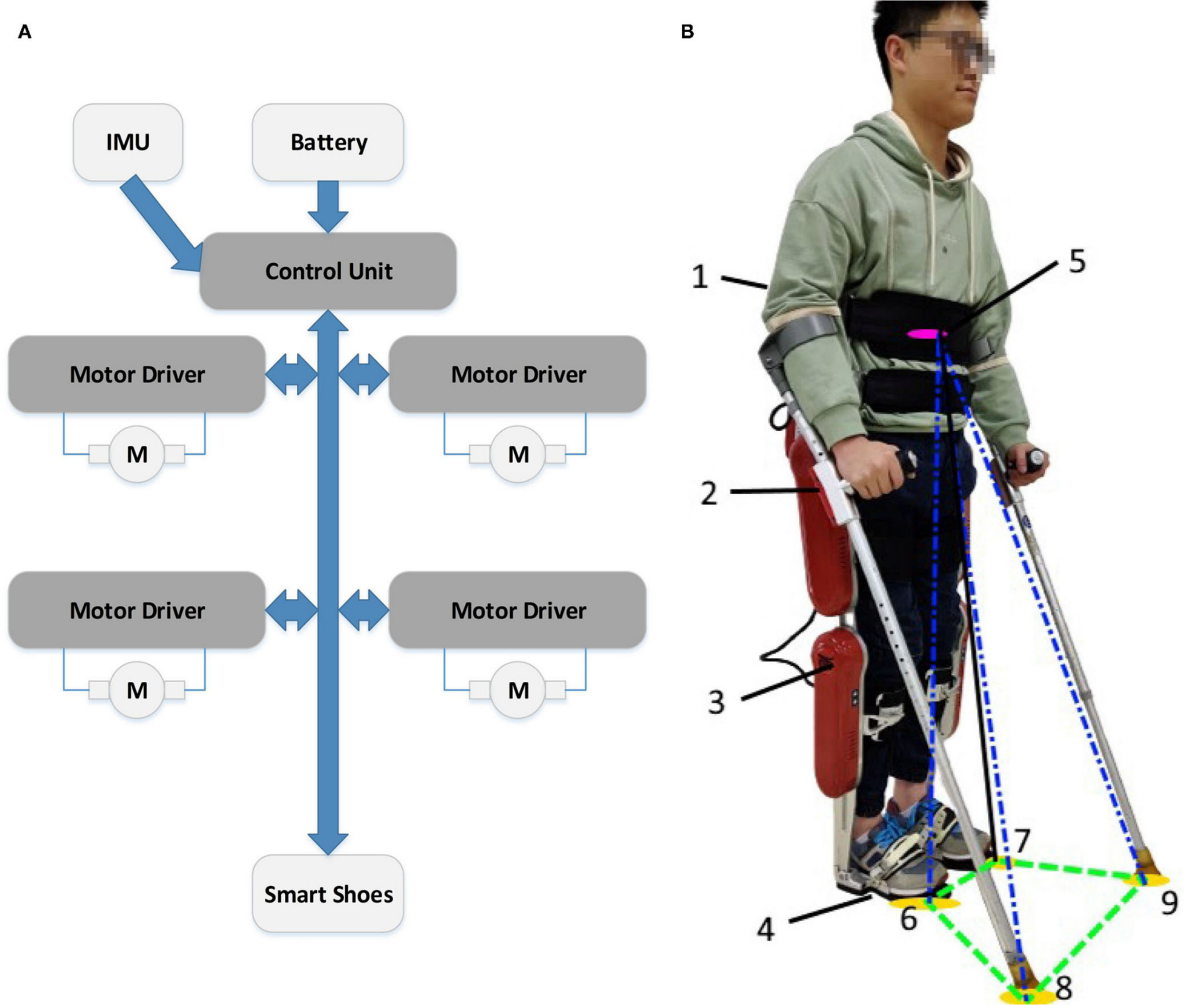
**(A)** is the basic functional diagram of the exoskeleton, and **(B)** is the human-exoskeleton system diagram. In the human-exoskeleton system, the pilot could stand like this with the exoskeleton. To collect the system's safety status, a lot of sensors are installed in the exoskeleton. 1: An Inertial Measurement Unit (IMU) in the backpack of the exoskeleton to sense the attitude and velocity of the pilot's upper body to estimate the position of the system's XCoM labeled as 5. 2: The IMU and pressure sensor to sense whether the crutch is in contact with the ground and the corresponding posture of the crutches, which could help to calculate part of BoS labeled as 8 and 9. 3: The angle encoder module to get the joint angles of the exoskeleton to get the position according to the forward kinematics module. 4: The pressure sensor under the foot to detect whether the foot touches the ground and estimate part of BoS labeled as 6 and 7.

After validating the ESPI in the human-exoskeleton simulation, the balance control strategy will be verified by the human-exoskeleton system shown in Figure 11. The experiments are carried out on the exoskeleton system AssItive DEvice for paRalyzed patient (AIDER), and its basic functional diagram is shown in Figure 11A. AIDER consists of a computer-based control unit, battery, IMUs, four motor units, and smart shoes with pressure sensors. The battery will provide power for this system, the control unit could process the data of the system and generate gaits, the motor units receive and execute the control signals with the joint angles uploaded, the smart shoes provide support and feedback on the foot pressure, and the IMUs could offer the attitude, angular velocity, and angular acceleration. During the experiments, the pilot is tightly connected to the exoskeleton through ties; thus, the attitude, velocity and acceleration estimated by the IMU labeled as 1 in Figure 11B could represent this status of the pilot's upper body. Furthermore, the attitude and pressure of the sticks and joint angles could be obtained by the sensors labeled 2 and 3, respectively in Figure 11B.

**Figure 12:**
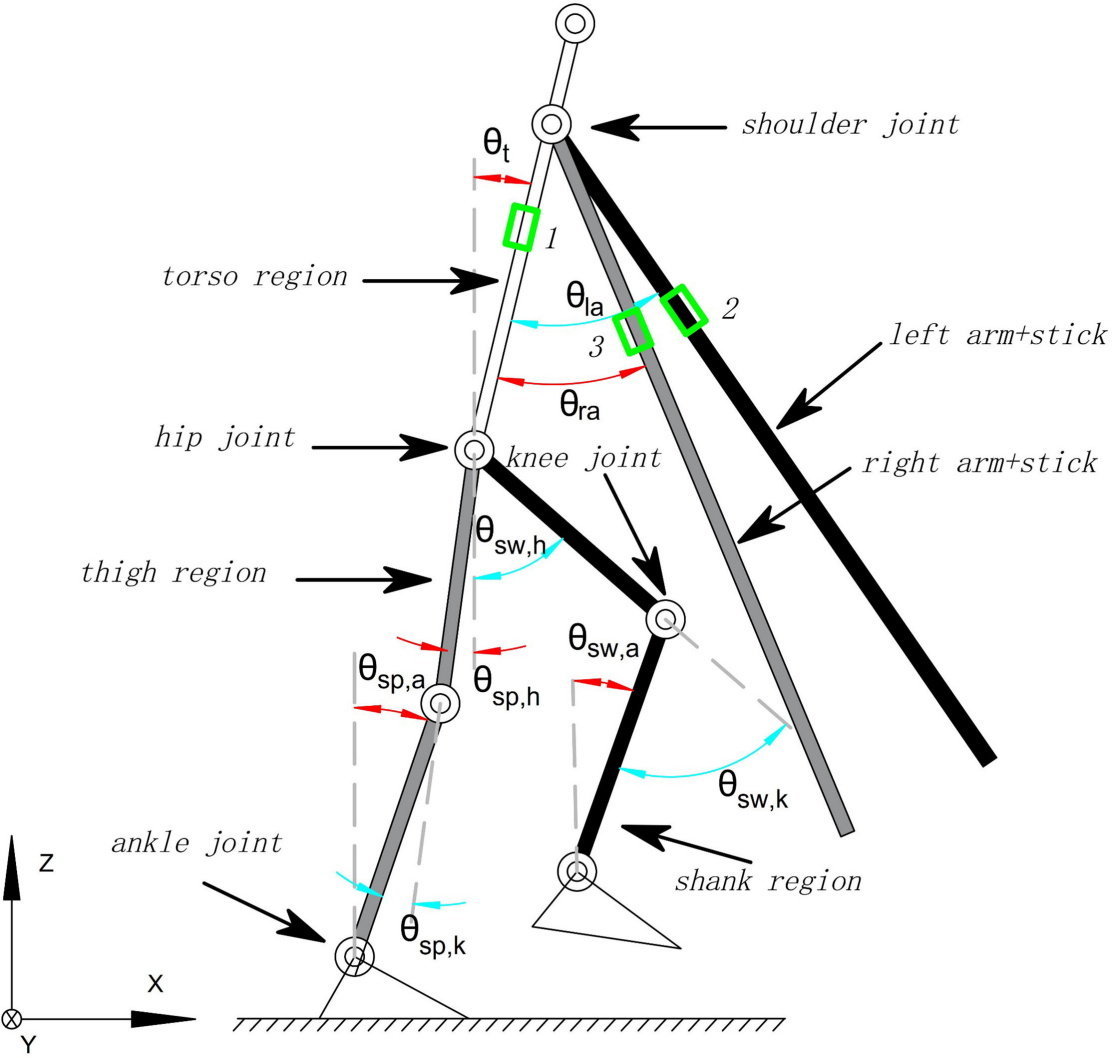
Human-exoskeleton model with sticks. It consists of the torso, arms, legs, and feet, which are linked by shoulder joints, hip joints, knee joints, and ankle joints. The human arms connected to the sticks are defined as left arm+stick and right arm+stick, respectively. The joint angles of lower limbs are defined in the model: the hip angles of support leg and swing leg θ_*sp,h*_, θ_*sw,h*_, the knee joint angles of support leg and swing leg θ_*sp,k*_, θ_*sw,k*_, and the ankle joint angles of support leg and swing leg θ_*sp,a*_, θ_*sw,a*_. They are measured by the encoders installed on the joints of the exoskeleton. Additionally, the upper limbs' joint angles are defined: the torso inclination angle θ_*t*_, the shoulder angles corresponding to the movements of the arms and sticks of left and right in the x-axis direction are θ_*lax*_ and θ_*rax*_. In contrast, the shoulder angles resulted from the movements in the y-axis direction are θ_*lay*_ and θ_*ray*_. Also, the length of the regions are defined as follows: *l*_*to*_: the length of torso, *l*_*as*_: the length of left/right arm and stick, *l*_*th*_: the length of the thigh, *l*_*sh*_: the length of the shank, and *h*_*f*_: the height of the foot. The IMUs are installed at the torso, the arms and sticks of left and right labeled 1, 2, 3, and the length from label 1 to the hip joint is defined as *l*_*com*_.

To make it clear, we presented the human-exoskeleton model shown in Figure 12. The joint angles and links are all defined in it. The position of the support foot is *P*_*sp,f*_, and it could be the origin of the coordinate system shown in Equation (38). Additionally, the position of other joints could be obtained by the forward kinematics with the joint angles and the length. All the joint angles like θ_*sp,h*_, θ_*sp,k*_, θ_*sp,a*_, θ_*sw,h*_, θ_*sw,k*_, and θ_*sw,a*_ could be measured directly by the encoders installed in the exoskeleton, the length of the links like *l*_*to*_, *l*_*th*_, *l*_*as*_, *l*_*sh*_, *h*_*f*_, and *l*_*com*_ could be measured before experiments.


(38) $$\begin{array}{l} P_{sp,f}={\left[\begin{array}{c} x_{sp,f}\\ y_{sp,f}\\ z_{sp,f} \end{array}\right]}^{T}={\left[\begin{array}{c} 0\\ -\frac{w}{2}\\ 0 \end{array}\right]}^{T} \end{array}$$

in which, ω is the width of the shoulder. The position of support and swing hip *P*_*sp,h*_, *P*_*sw,h*_ are shown in Equations (39, 40).


(39) $$\begin{array}{l} P_{sp,h}={\left[\begin{array}{c} x_{sp,h}\\ y_{sp,h}\\ z_{sp,h} \end{array}\right]}^{T}={\left[\begin{array}{c} x_{sp,f}+l_{sh}*\sin\theta_{sp,a}+l_{th}*\sin\theta_{sw,h}\\ -\frac{w}{2}\\ z_{sp,f}+h_{f}+l_{sh}*\cos\theta_{sp,a}+l_{th}*\cos\theta_{sw,h} \end{array}\right]}^{T} \end{array}$$

(40) $$\begin{array}{l} P_{sw,h}={\left[\begin{array}{c} x_{sw,h}\\ y_{sw,h}\\ z_{sw,h} \end{array}\right]}^{T}={\left[\begin{array}{c} x_{sp,h}\\ \frac{w}{2}\\ z_{sp,h} \end{array}\right]}^{T} \end{array}$$

Then, based on the position of the swing hip joint and the joint angles, the position of swing foot *P*_*sw,f*_ could be calculated in Equation (41).


(41) $$\begin{array}{l} P_{sw,f}={\left[\begin{array}{c} x_{sw,f}\\ y_{sw,f}\\ z_{sw,f} \end{array}\right]}^{T}\\ \;={\left[\begin{array}{c} x_{sw,h}+l_{th}*\sin\theta_{sw,h}+l_{sh}*\sin\left(\theta_{sw,h}-\theta_{sw,k}\right)\\ \frac{w}{2}\\ z_{sw,h}-l_{th}*\cos\theta_{sw,h}-l_{sh}*\cos\left(\theta_{sw,h}-\theta_{sw,k}\right)-h_{f} \end{array}\right]}^{T} \end{array}$$

After getting *P*_*sp,f*_ and *P*_*sw,f*_, we are going to obtain the position of the contact points between the left and right stick and the ground, which are expressed as *P*_*la*_ and *P*_*r*_*a*__. Before it, the position of left and right shoulder *P*_*l,sh*_, *P*_*r,sh*_ could be obtained.


(42) $$\begin{array}{l} P_{l,sh}={\left[\begin{array}{c} x_{l,sh}\\ y_{l,sh}\\ z_{l,sh} \end{array}\right]}^{T}={\left[\begin{array}{c} x_{sp,h}+l_{to}*\sin\theta_{t}\\ \frac{w}{2}\\ z_{sp,h}+l_{to}*\cos\theta_{t} \end{array}\right]}^{T} \end{array}$$

(43) $$\begin{array}{l} P_{r,sh}={\left[\begin{array}{c} x_{r,sh}\\ y_{r,sh}\\ z_{r,sh} \end{array}\right]}^{T}={\left[\begin{array}{c} x_{l,sh}\\ -\frac{w}{2}\\ z_{l,sh} \end{array}\right]}^{T} \end{array}$$

Also, the position of CoM could be expressed as below:


(44) $$\begin{array}{l} P_{com}={\left[\begin{array}{c} x_{com}\\ y_{com}\\ z_{com} \end{array}\right]}^{T}={\left[\begin{array}{c} x_{sp,h}+l_{com}*\sin\theta_{t}\\ 0\\ z_{sp,h}+l_{com}*\cos\theta_{t} \end{array}\right]}^{T} \end{array}$$

in which, *l*_*com*_ is the length between the hip joint and the IMU on the back, θ_*t*_ is the inclination angle of the torso, which could be estimated by the IMU labeled 1 in Figure 12.


(45) $$\begin{array}{l} P_{la}={\left[\begin{array}{c} x_{la}\\ y_{la}\\ z_{la} \end{array}\right]}^{T}={\left[\begin{array}{c} x_{l,sh}+l_{as}*\sin\theta_{lax}\\ \frac{w}{2}+l_{as}*\sin\theta_{lay}\\ 0 \end{array}\right]}^{T} \end{array}$$

(46) $$\begin{array}{l} P_{ra}={\left[\begin{array}{c} x_{ra}\\ y_{ra}\\ z_{ra} \end{array}\right]}^{T}={\left[\begin{array}{c} x_{r,sh}+l_{as}*\sin\theta_{rax}\\ -\frac{w}{2}-l_{as}*\sin\theta_{ray}\\ 0 \end{array}\right]}^{T} \end{array}$$

When the sticks contact the ground, the positions could be calculated according to Equations (45, 46). Among them, the angles θ_*lax*_, θ_*lay*_, θ_*rax*_, and θ_*rax*_ are estimated by the IMUs labeled 2 and 3, respectively.

All the IMUs used in this study would get the velocity, acceleration, and attitude with the help of Digital Motion Processor developed by InvenSense. Before being installed on the human-exoskeleton system, each IMU will be calibrated. First, the IMU will be placed still and horizontally; then, the offset value of each coordinate axis of the gyroscope and accelerometer will be saved so that the calibration function can be called to calibrate the sensor every time it turns on. Thus, the raw data of sensors are calibrated. After all the IMUs are installed, the system will first calibrate the IMU attitude angles. When the human-exoskeleton system stands upright, the data at this time will be regarded as the initial value θ_0_, and then the value at time *t* could be expressed as θ_*t*_. Finally, the angle change θ we need can be expressed by the following equation.


(47) $$\begin{array}{l} \theta =\theta_{t}-\theta_{0} \end{array}$$

**Figure 13:**
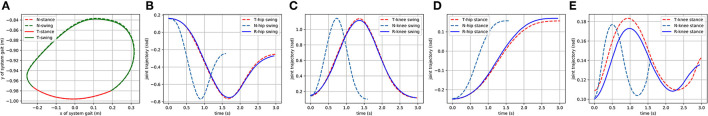
The temporal scaled gait and joint trajectories. The gait could be composed of the trajectories during stance and swing. For example, in **(A)**, the dotted lines are the normal ones used as reference labeled as N-stance and N-swing, and the solid ones are the temporal scaled marked as T-stance and T-swing. **(B–E)** The hip and knee joint trajectories of the normal, temporal, and actual system are labeled starting with N, T, and R, respectively.

**Figure 14:**
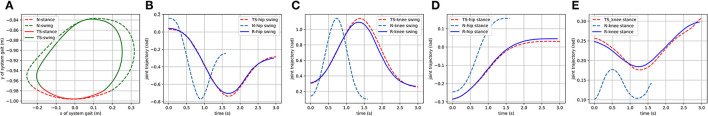
The temporal-spatial scaled gait and joint trajectories. In **(A)**, the dotted and the solid are the normal and temporal-spatial gaits, including stance and swing phase, and they are labeled as N-stance, N-swing, TS-stance, and T-swing, respectively. **(B–E)** The data of the actual system are the solid lines labeled starting with R, while the dotted ones are the normal and temporal-spatial joint trajectories marked beginning with N and TS, respectively. The errors of the trajectories between the feedback of the actual system and the planned are acceptable.

With the calibrated sensor data and equations above, the XCoM and BoS of ESPI could be estimated. The XCoM could be estimated with the sensor data of IMU labeled 1 in Figure 12 according to Equations (3) and (44). Meanwhile, with the help of joint angles and links' length, the BoS consisted of *P*_*sp,f*_, *P*_*sw,f*_, *P*_*la*_, and *P*_*ra*_ labeled as 5, 6, 7, and 8 in Figure 11B could be estimated with the help of Equations (38), (41), (45), and (46). So, the ESPI and the scaling parameters (τ_*s*_, τ_*t*_) for the gait generator are obtained according to the relationships shown in Figure 5. Once the parameters enter, the gait trajectory generator based on DMPs will work with the standard gait as a reference. Additionally, the new gaits could be planned by the gait generator based on the DMPs and executed in the exoskeleton system shown in Figures 13, 14. Finally, the joint trajectories of the new gait will be transformed by the inverse kinematics module and executed by the exoskeleton system.

When the safety status is not urgent, the τ_*t*_ could be bigger than before, and the gait trajectory could stay the same with the reduction of speed. The normal and temporal scaled gaits are shown in Figure 13A, while the hip and knee joint trajectories of normal, temporal scaled, and the feedback of the exoskeleton during are represented in Figures 13B–E. If the safety status changes and the stride length needs to be smaller than before, the τ_*t*_ could be bigger while the τ_*s*_ would be smaller than before. The new gait and joint trajectories could be shown in Figure 14 like the temporal ones above. Additionally, all the planned gaits are executed well by the exoskeleton system, with the errors between the planned trajectories and the actual execution ones meeting the requirements of the system, which proves the practicability of the ESPI and the balance control strategy.

## 4. Conclusions

To be able to evaluate the safety status of the human-exoskeleton system dynamically, this study proposed ESPI based on SPI with the following advantages: 1) it integrates dynamic information by introducing XCoM, 2) it can adjust the weight automatically by introducing the TTC as the urgency, and 3) it will reflect the safety status better through scalar and vector. By establishing a human-exoskeleton model and completing the LIP balance walking experiments in Gazebo, the effectiveness and sensitivity of ESPI have proved to be better than that of the SPI. Five different types of stability pyramid and the safety change direction of ESPI have been shown. Simultaneously, with the help of the ESPI, the balance control strategy for the human-exoskeleton based on DMPs is proposed. With the help of the scalar and vector values, the gait generator could tune different gaits to keep the system safe according to the spatial and temporal features of DMPs. Finally, the balance control strategy that combines ESPI and DMPs has been validated based on the human-exoskeleton system.

In the future, it will be important to explore the potential adaptability of ESPI to people of different weights and the balance control strategy on more active joints.

## Data Availability Statement

The original contributions presented in the study are included in the article/supplementary material, further inquiries can be directed to the corresponding author.

## Ethics Statement

The studies involving human participants were reviewed and approved by the Ethics Committee of the University of Electronic Science and Technology of China. The patients/participants provided their written informed consent to participate in this study. Written informed consent was obtained from the individual(s) for the publication of any potentially identifiable images or data included in this article.

## Author Contributions

FX contributed to the conception and design of the study, performed the experiments, and drafted the manuscript. WY contributed to data collection. JQ and HC guided the writing of the study. All the authors contributed to manuscript revision, read, and approved the submitted version.

## Funding

This work was supported by the National Key Research and Development Program of China (No. 2018AAA0102505), the Sichuan Major Scientific and Technological Special Project (No. 2018GZDZX0037), and National Natural Science Foundation of China (NSFC) (No. 62003073).

## Conflict of Interest

JQ was employed by company Buffalo Robot Technology (Chengdu) Co., Ltd. The remaining authors declare that the research was conducted in the absence of any commercial or financial relationships that could be construed as a potential conflict of interest.

## Publisher's Note

All claims expressed in this article are solely those of the authors and do not necessarily represent those of their affiliated organizations, or those of the publisher, the editors and the reviewers. Any product that may be evaluated in this article, or claim that may be made by its manufacturer, is not guaranteed or endorsed by the publisher.
